# Brain Abscess as Severe Presentation of Specific Granule Deficiency

**DOI:** 10.3389/fped.2020.00117

**Published:** 2020-04-22

**Authors:** Maria Leszcynska, Bhumika Patel, Matthew Morrow, Wil Chamizo, Gerald Tuite, David M. Berman, Kevin Potthast, Amy P. Hsu, Steven M. Holland, Jennifer W. Leiding

**Affiliations:** ^1^Pediatric Residency Training Program, Johns Hopkins-All Children's Hospital, St. Petersburg, FL, United States; ^2^Division of Allergy and Immunology, Department of Pediatrics, University of South Florida, St. Petersburg, FL, United States; ^3^Immunology Lab, Department of Pathology and Laboratory Medicine, Johns Hopkins All Children's Hospital, St. Petersburg, FL, United States; ^4^Pediatric Neurosurgery, Johns Hopkins All Children's Hospital, St. Petersburg, FL, United States; ^5^Pediatric Infectious Diseases, Johns Hopkins All Children's Hospital, St. Petersburg, FL, United States; ^6^Department of Radiology, Johns Hopkins All Children's Hospital, St. Petersburg, FL, United States; ^7^Laboratory of Clinical Immunology and Microbiology, National Institute of Allergy and Infectious Diseases, National Institutes of Health, Bethesda, MD, United States

**Keywords:** DHR assay, specific granule deficiency, neutrophil, brain abscess in children, phagocyte

## Abstract

Severe invasive infections such as brain abscess in a child should prompt an immune evaluation. Specific granule deficiency (SGD) is a rare morphologic neutrophil granular defect characterized by reduced granules within neutrophils, absence of granule proteins, and bilobed nuclei. Patients are susceptible to invasive bacterial infections and *Candida* infections. Mutations in CCAT/enhancer binding protein epsilon (C/EBP-ε) are the most commonly described cause of SGD. The dihydrorhodamine assay is a quantitative and qualitative functional test that determines the oxidative burst and killing potential of neutrophils. Herein, we describe two brothers with specific granule deficiency. The index patient had a history of cellulitis twice in the first year of life and then presented at 13 months age with fever, leukocytosis, and right sided weakness. A large space occupying brain abscess was diagnosed. He underwent surgical drainage and cultures yielded *Staphylococcus aureus*. This infection prompted his diagnosis. His older brother had also been healthy but too had had several episodes of cellulitis. His brother too was diagnosed with SGD when family genetic screening was performed. Evaluation of the index patient included a peripheral smear that showed absent neutrophil granule presence. Forward and side scatter of whole blood via flow cytometry revealed a loss of granularity of neutrophils. A DHR was performed to rule out functional killing defects. After stimulation with PMA, neutrophils from the index patient displayed three distinct patterns, two with abnormal oxidase production, and two with reduced function. Both patients were ultimately diagnosed with SGD and remain on lifelong anti-bacterial prophylaxis. Diagnosis of SGD relies on establishing reduced or absent granularity within neutrophils. Lifelong anti-bacterial and anti-fungal prophylaxis is indicated. Hematopoietic cell transplantation has also been curative.

## Introduction

Neutrophil granules are a first line of host defense. Mature neutrophils contain a number of pre-formed receptors and microbicidal proteins within granules. Primary (azurophilic) granules contain myeloperoxidase. Secondary granules contain lactoferrin and transcobalmin-1 and tertiary granules contain neutrophil elastase.

Neutrophil specific granule deficiency (SGD) is a rare neutrophil defect in which neutrophils lack specific granules. Patients often present in the first few years of life and have a susceptibility to severe invasive pyogenic infections from *Staphylococcus aureus, Candida albicans*, and *Pseudomonas aeruginosa*. There are few cases reported of patients with SGD (1–6). Infections can range from recurrent skin abscesses to severe invasive bacterial infections. Sino-pulmonary bacterial infections are also common. Central nervous system infection has been reported in only one case: a 25 years old man who had bacterial meningitis complicated by a subdural empyema as a child (4). Hematopoietic cell transplant has been successful in treating SGD in at least 1 patient (7).

Mutations in CCAT/enhancer binding protein epsilon (C/EBP-ε), have been described as the major cause of SGD (2, 3). C/EBP-ε is a critical transcription factor for myeloid development, more specifically for neutrophil differentiation past the promyelocytic stage. It regulates transcription of the components of secondary and tertiary granules within neutrophils and eosinophils (8–10). Neutrophils from patients with SGD lack expression of specific granule proteins including bactericidal permeability increasing protein (BPI) and defensins or human neutrophil peptides (11). Additionally, they show an increased expression of members of the linker of nucleoskeletan and cytoskeleton complex that control nuclear shape (5). Functional deficits have also been described in SGD neutrophils. Neutrophil chemotaxis and killing of *S. aureus in vitro* have been shown to be impaired (3, 8). Oxidative burst of SGD neutrophils is variable. Reduced activity of the NADPH oxidase after stimulation with zymosan (2, 12, 13) has been described, although the NADPH oxidase complex appears to still be present with normal intra phagocytic vacuole oxidative burst (5).

Diagnosis of SGD is based on the abnormal appearance of neutrophils, which can easily be accomplished with a peripheral smear. Characteristic features include an atypically bilobed nucleus and abnormal granule numbers and contents.

Herein, we present a toddler diagnosed with specific granule deficiency after presentation with a brain abscess and abnormal dihydrorhodamine assay. Written informed consent was obtained from the parents of the subject for publication of this case report.

## Patient and Methods

### Case Presentation

**Figure 1:**
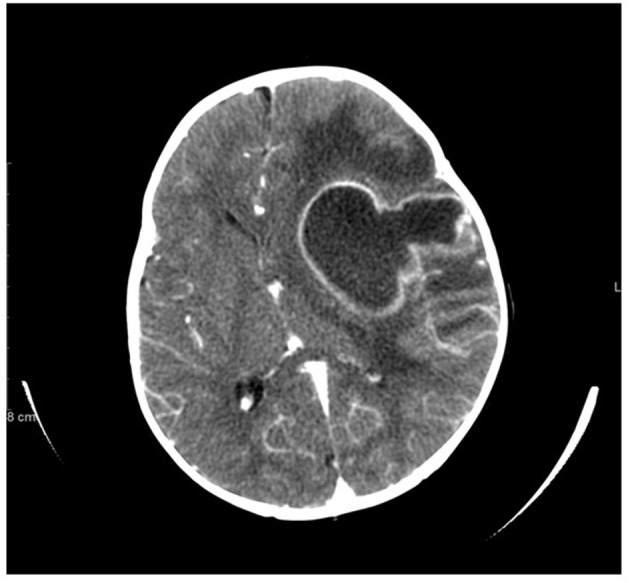
Non-contrast CT scan of brain at presentation demonstrates large left cavitary abscess with inflammatory sediment, left hemispheric edema, ventricular compression, severe mass effect, and midline shift.

A 13-months old Sudanese male presented with low grade fever and irritability. He was diagnosed with acute otitis media and placed on oral amoxicillin. Fever and irritability initially resolved but on day 7 of amoxicillin, fever (102°F) returned along with leukocytosis (20,000 cells/μL). Intramuscular ceftriaxone was given but within 24 h, he had developed vomiting, right arm weakness, worsening irritability, and persistent fever. Because of the onset of neurologic symptoms, a CT scan of the brain was performed and showed a large left parietal brain mass with midline shift and cerebral edema (Figure 1). He underwent emergent left parietal craniotomy and evacuation of the brain abscess. Cultures yielded methicillin-susceptible *Staphylococcus aureus* (MSSA). He was treated with intravenous ceftriaxone for 6 weeks. He had resolution of the infection and complete resolution of neurologic symptoms. His previous history included a 36-weeks' gestation to consanguineous Sudanese parents. He was monitored in the neonatal intensive care unit for the first 4 days of life due to low birth weight. In the first year of life, he had two episodes of cellulitis, involving the lower leg and upper eyelid. Both episodes resolved with oral antibiotics. This patient's 4-year-old brother also had recurrent cellulitis and cutaneous abscesses at sites of minimal trauma for a toddler (upper back, face, abdomen).

### Dihyrdrorhodamine Assay

**Figure 2:**
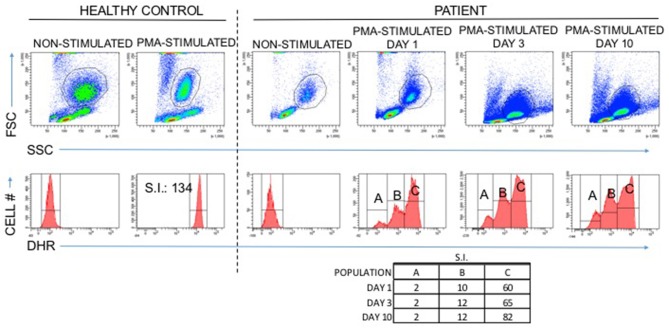
Abnormal DHR results in a patient with Specific Granule Deficiency: A normal DHR response is at least an 85-fold increase in mean fluorescence intensity when granulocytes are stimulated with PMA *in vitro* compared to non-stimulated granulocytes (healthy control). The patient with specific granule deficiency produced an abnormal result with three distinct populations, two of which failed to reach the 85-fold increase associated with normal granulocyte oxidative burst.

**Figure 3:**
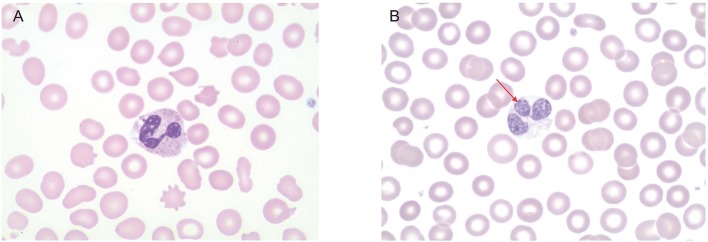
Peripheral smear. **(A)** Healthy control. Neutrophils have normal multi-lobed nucleus and abundance of granules in the cytoplasm. **(B)** Patient's peripheral smear. Arrow indicates abnormally lobed nuclei. There is also a paucity of granules in the cytoplasm.

Because of the early onset, severity, and invasiveness of this infection, an immune evaluation was performed. Immunoglobulin subsets and lymphocyte subset quantities were normal. With his history of cutaneous abscesses and presentation with a life threatening invasive abscess, a neutrophil defect was highly suspected. During acute infection, the absolute neutrophil count (ANC) increased to as high as 23,800 cells/μL. Once the infection had resolved ANC's were normal (2,480–3,730 cells/μL, reference range 1,500–8,500 cells/μL). A dihydrorhodamine (DHR) assay was ordered to evaluate for chronic granulomatous disease. Granulocytes were isolated from whole blood via flow cytometry with results that were inconclusive (Figure 2). A DHR was performed again 48 h and then 1 week later with similar results. At all three time points, three distinct populations were isolated, two of which had abnormal oxidative burst detected. In addition, analysis of the scatter plot (Figure 2) showed that the granulocyte population could not be easily separated from the lymphocyte population based on forward and side scatter. The DHR was repeated on two more occasions with similar results. Since granularity is a major feature that differentiates granulocytes from lymphocytes, our patient's scatter plot indicated a loss of granularity in granulocytes, further focusing our attention on a neutrophil granule defect. A peripheral smear showed normal appearing neutrophils with a paucity of specific granules (Figures 3A,B) and bone marrow aspiration revealed myeloid hypoplasia with reduced quantity of mature granulocytes. A homozygous non-sense mutation was found in *CEBP*ε, c.403 C>T p.R135X, confirming the diagnosis of specific granule deficiency.

## Discussion

Neutrophils play an important role in innate and adaptive immune responses against certain micro-organisms. Abnormalities of neutrophil development, quantities, migration, phagocytosis, and bactericidal activity have been described. The initial evaluation of neutrophil disorders includes serial measures of absolute neutrophil count, examination of a peripheral smear to examine neutrophil morphology and granule content, and a DHR assay to examine neutrophil superoxide production (14).

Following phagocytosis, primary azurophilic, secondary specific, tertiary gelatinase, and secretory vesicles fuse with phagosomes releasing micobicidal proteins that cause direct micro-organism killing (3). CCAAT/enhancer-binding proteins (C/EBP) are a family of six transcription factors which are involved with a number of cellular processes including cellular differentiation (15). C/EBPε is specifically expressed in myeloid cells and is responsible for the terminal differentiation of granulocyte progenitors (3). Defects in C/EBPε block transition of neutrophils from the promyelocyte to myelocyte stage. Primary azurophilic proteins are produced in myeloblasts and promyeloblasts, secondary specific granule proteins in myelocytes and metamyelocytes, and tertiary gelatinase in bands and polymorphonuclear neutrophils. In SGD caused by C/EBPε mutations, both secondary and tertiary granules are absent (3). Lactoferrin, a strong bacteriostatic factor and the primary enzyme produced in specific granules, is deficient in SGD patients (1). Other cytotoxic molecules such as lysozyme and defensins are also absent in SGD patients. In addition to these abnormalities in neutrophil morphology, neutrophils from SGD patients show abnormalities in migration and reduced *in vitro* killing of *S. aureus* (2, 5, 13). Oxidative metabolism of SGD neutrophils is more variable and may be linked to the infection or clinical status of the patient, making functional tests of oxidative burst production difficult to interpret. Oxidative burst has been enhanced (13) or normal (2, 5) after neutrophil stimulation with f-met-leu-phe (FMLP), normal (5), or reduced (2) after stimulation with PMA, and reduced after stimulation with zymosan (2, 12, 13). In the case of our patient, after stimulation with PMA our patient's neutrophils exhibited an abnormal oxidative burst via a DHR assay and so the DHR was useful in establishing the patient's diagnosis.

Abnormalities in other cells have also been described. SGD eosinophils lack granules that contain eosinophilic cationic protein, eosinophil-derived neurotoxin, and major basic protein (16). Monocytes from SGD patients are morphologically abnormal and express decreased levels of the monocyte-specific enzyme non-specific esterase (17).

The clinical course and presentation of SGD patients has not been well-established due to the rarity of the disease. Clinical manifestations of SGD can include chronic, often severe cutaneous infections. Patients may also develop severe invasive bacterial infections. SGD is readily diagnosed by examining a peripheral smear, negating the need for functional testing to establish a diagnosis. Granulocytes will lack specific granules and neutrophils will be difficult to distinguish from eosinophils because of their lack of eosinophilic granules. Flow cytometric analysis of neutrophil granule proteins may aid in rapidly confirming the diagnosis. Severe deficiencies in defensins, lactoferrin, BPI, human cathelicidin antimicrobial peptide 18 (hCAP18), and neutrophil gelatinase-associated lipocalin (NGAL) confirm SGD (18). Finally, molecular analysis of C/EBPε definitively confirms the diagnosis. However, there are also C/EBPε negative cases, indicating that C/EBPε is not the only gene governing specific granulogenesis.

Treatment consists mainly of prophylactic antibiotics such as trimethoprim-sulfamethoxazole to prevent infections. When infections occur, debridement and intravenous antibiotics may be necessary. Successful hematopoietic cell transplant has been performed in at least one patient using a busulfan based conditioning regimen (7).

Our patient presented with a severe life threatening brain abscess. After diagnosis of SGD, he was placed on TMP-SMX prophylaxis and has thrived. He is currently a developmentally normal 4-year-old who has had no cutaneous or severe infections since initiation of prophylaxis. Transplant evaluation showed no unrelated donors. His older brother had the same homozygous mutation in C/EBPε and is also receiving prophylaxis with TMP-SMX and is thriving. Anti-fungal prophylaxis was not used based on lack of any previous fungal infections, including mucocutaneous infections, in either patient, and parent discretion.

## Conclusions

Severe invasive bacterial infections such as a brain abscess in a child should warrant an immune evaluation. Invasive infection with *Staphylococcus aureus* indicates a possible neutrophil abnormality. SGD is a rare cause of immune deficiency but can be easily suspected by examination of a peripheral smear. Functional testing of neutrophils, including the DHR, may also be abnormal in SGD. Diagnosis and proper anti-microbial prophylaxis prevent infections and disease-related morbidity.

## Data Availability Statement

The raw data supporting the conclusions of this article will be made available by the authors, without undue reservation, to any qualified researcher.

## Ethics Statement

Written informed consent was obtained from the minor(s)' legal guardian/next of kin, for the publication of any potentially identifiable images or data included in this article.

## Author Contributions

ML, BP, and JL wrote the manuscript. MM and WC performed assays and edited the manuscript. AH and SH performed genetic testing and edited the manuscript. GT, DB, KP, and JL provided patient care, conceptualized the project, and edited the manuscript.

## Conflict of Interest

The authors declare that the research was conducted in the absence of any commercial or financial relationships that could be construed as a potential conflict of interest.
